# 14,15-Secopregnane-Type Glycosides with 5α:9α-Peroxy and Δ^6,8(14)^-diene Linkages from the Roots of *Cynanchum stauntonii*

**DOI:** 10.3390/molecules22060860

**Published:** 2017-05-23

**Authors:** An-Jun Deng, Jin-Qian Yu, Zhi-Hong Li, Lin Ma, Zhi-Hui Zhang, Hai-Lin Qin

**Affiliations:** 1 State Key Laboratory of Bioactive Substance and Function of Natural Medicines, Institute of Materia Medica, Chinese Academy of Medical Sciences and Peking Union Medical College, Beijing 100050, China; denganjun@imm.ac.cn (A.-J.D.); yujinqian87528@126.com (J.-Q.Y.); zhl@imm.ac.cn (Z.-H.L.); malin@imm.ac.cn (L.M.); zhangzhihui@imm.ac.cn (Z.-H.Z.); 2 Shandong Analysis and Test Center, Shandong Academy of Sciences, Jinan 250014, Shandong, China

**Keywords:** *Cynanchum stauntonii*, Asclepiadaceae, 14,15-secopregnane-type glycoside, stauntogenin G, 5α:9α-peroxy linkage, stauntosides UA, UA_1_, and UA_2_, structure elucidation

## Abstract

Three new 14,15-secopregnane-type glycosides, stauntosides UA, UA_1_, and UA_2_, were isolated from the roots of *Cynanchum stauntonii*. The three compounds share the first reported and same basic structural features of 3β-hydroxy-14:16,15:20,18:20-triepoxy-5α:9α-peroxy-14,15-secopregnane-6,8(14)-diene named as stauntogenin G as the aglycones. The structures of the new compounds were characterized on the basis of extensive spectroscopic analyses, mainly 1D and 2D NMR and MS methods and chemical analysis. The isolation and identification of the new compounds graced the structural diversity of pregnane-type steroids from *C. stauntonii*.

## 1. Introduction

**Figure 1:**
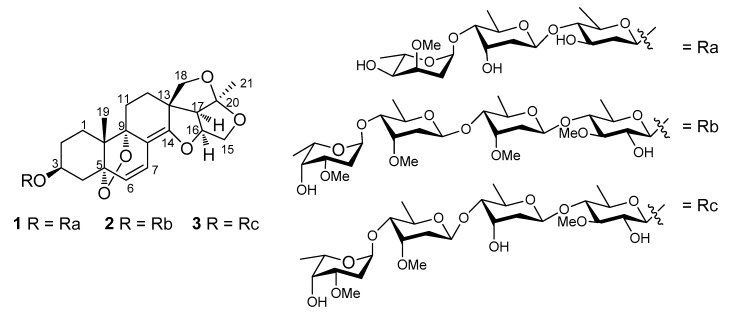
Structures of compounds **1**–**3**.

C_21_-Pregnane-type natural organic compounds possess the usual skeleton of 17-ethyl-10,13-dimethylhexadecahydro-1*H*-cyclopenta[*a*]phenanthrene on the systematic nomenclature. The natural resource of this class of steroids is very affluent in the plant world, with both sugar-free and glycosidated pregnane-type steroids having been isolated by many researchers through phytochemical methods. In addition to the usual four-ring C_21_-pregnane-type skeleton, there are also several unusual skeletons, such as the 8,14-seco-C_21_-pregnane-type, 14,15-seco-C_21_-pregnane-type, and 13,14:14,15-diseco-C_21_-pregnane-type skeletons, all of these usual and unusual skeletons possessing multiple stereogenic centers and other structural diversities [1,2,3]. It is well known that the *Cynanchum* species of the Asclepiadaceae family are very rich in C_21_-steroids, with the unusual 14,15-seco-C_21_-pregnane-type and 13,14:14,15-diseco-C_21_-pregnane-type skeletons being most often discovered in previous investigations [4,5,6,7,8,9,10,11]. It is also well known in natural medicinal chemistry that, in addition to the structural diversity, C_21_-steroids are one class of biologically active compounds, with multiple bioactivities being reported [7,9,10,11,12]. Especially, our group reported that some steroidal glycosides isolated from *C. stauntonii* (Decne.) Schltr. ex Levl., a perennial medicinal herb naturally growing in the south-central region of China, showed anti-inflammatory activity [3]. This finding provided evidence supporting the application of *C. stauntonii* in some traditional medicine systems to treat inflammations [3,13,14]. Following the isolation and identification of several steroidal glycosides from the roots of *C. stauntonii* sharing the first reported aglycones of 8α:14α,14:16,15:20,18:20-tetraepoxy-14,15-secopregn-6-ene-3β,5α,9α-triol or its 5α:9α-peroxy bridge structure [3], our ongoing searches for new steroids in the same subjects lead to the isolation of three new steroidal glycoside, stauntosides UA, UA_1_, and UA_2_ (**1**–**3**) (Figure 1). Structural identification affirmed that stauntosides UA, UA_1_, and UA_2_ shared an aglycone of 3β-hydroxy-5α:9α-peroxy-14:16,15:20,18:20-triepoxy-14,15-secopregnane-6, 8(14)-diene, similar to, but somewhat different from the aforementioned first reported aglycones. Thus, the three new steroids were regarded as another subcategory of 14,15-secopregnane-type steroids. In order to grace the structural diversity of pregnane-type steroids in *C. stauntonii*, this paper describes the isolation and structure elucidation of these new compounds.

## 2. Results and Discussion

The roots of *C. stauntonii* were extracted with 95% EtOH. The 95% EtOH extract was concentrated and partitioned using petroleum ether and EtOAc. The EtOAc-soluble fraction was separated using multiple column chromatographies and preparative HPLC. As a result, three new compounds reported herein were yielded, all as white amorphous powders. All three compounds showed positive Libermann-Burchard and Keller-Kiliani reactions, suggesting their glycosidated steroids or triterpenoids categories with 2-deoxysugars in their sugar moieties [2,15]. The category of steroidal glycosides was determined according to their shared common features in the NMR spectra. The three or four distinguishable anomeric proton signals of sugars in the ^1^H-NMR spectra indicated the presence of corresponding sugar moieties (Table 1). When putting off the carbon signals of the three or four hexose units, all three compounds left twenty-one carbons from the C_21_-pregnane moieties (Table 1 and Table 2). All three compounds showed two singlets of methyl groups in their ^1^H-NMR spectra. The δ values of the relatively lower-field singlet of methyl groups in each compound, i.e., δ_H_ 1.54 for all three compounds, along with the carbon signals at δ_C_ 118.4, 119.6, and 118.2 in the ^13^C-NMR spectra for compounds **1**–**3**, respectively, suggested that they belonged to the unusual 14,15-seco- (or 13,14:14,15-diseco-) C_21_-pregnane-type steroids. These singlets of methyl groups in the ^1^H-NMR spectra were born of Me-19 and 21 of the C_21_-pregnane skeleton, respectively. These carbon signals are the typical features of a dioxygenated secondary carbon-20 structure and they were confirmed by the correlations from Me-21 to C-17 and 20 in the respective HMBC spectrum of the three compounds. No carbonyl carbon was present in the ^13^C-NMR spectra of all three compounds, assigning them to be the 14,15-seco-C_21_-pregnane-type steroids [2,3,7].

**Figure 2:**
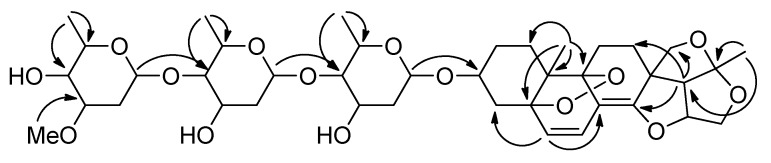
Key HMBC correlations (H→C) of **1**.

**Figure 3:**
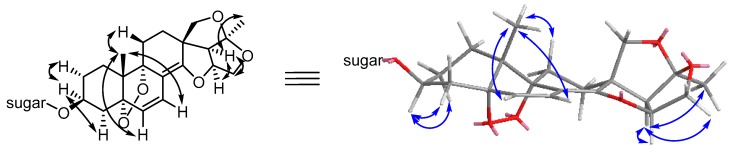
Key NOE correlations (H↔H) in the aglycone moiety of **1**.

Compound **1** possessed a molecular formula of C_40_H_58_O_15_ according to its ^13^C-NMR spectroscopic data and the HRESIMS (positive ion mode) data for the protonated molecular ion at *m*/*z* 779.3861 and sodium adduct molecular ion at *m*/*z* 801.3694, indicating a hydrogen deficiency index of twelve. This molecular formula has one fewer oxygen atom than that of stauntoside V_3_, a 14,15-secopregnane-type glycoside with the aglycone of stauntogenin F (3β-hydroxy-8α:14α,14:16,15:20,18:20-tetraepoxy-5α:9α-peroxy-14,15-secopregn-6-ene) previously isolated by our group from *C. stauntonii* [3]. Its IR spectrum displayed absorption bands for hydroxy (3440 cm^−1^) and olefinic (1681 cm^−1^) functionalities, among others. Acid hydrolysis, along with derivatization and GC analysis, indicated the presence of d-canaropyranose, d-digitoxopyranose, and l-cymaropyranose in a 1:1:1 ratio. The entire ^1^H and ^13^C-NMR spectroscopic data for **1** are given in Table 1 and Table 2, respectively. A detailed comparison of NMR data between **1** and stauntoside V_3_ showed that they were very similar. In the ^1^H-NMR spectrum, all the signals for **1** were nearly superimposable on their counterparts in stauntoside V_3_, the coupling constants of the three anomeric protons provided evidence that two monosaccharides shared β-glucosidic bonds and one possessed an α*-*glucosidic bond. In the ^13^C-NMR spectrum, the primary difference was in the replacement of the signals for an oxygenated tertiary carbon at δ_C_ 70.8 (s, C-8) and an dioxygenated secondary carbon at δ_C_ 98.9 (s, C-14) in the known compound by the signals of an olefinic quaternary carbon at δ_C_ 110.4 (C-8) and an oxygenated olefinic tertiary carbon at δ_C_ 156.8 (s, C-14) in **1**. The rest of the carbons of compound **1** showed full accordance with categories of carbon types with their respective counterparts in stauntoside V_3_. The conjugated highfield shifts of C-6 and C-7 by Δ_δ_ –10.9 and −2.7, respectively, in compound **1** compared with stauntoside V_3_ were evident, which proposed a double bond linkage between C-8 and C-14. A highfield shift of Δ_δ_ –6.2 for C-10 compared with stauntoside V_3_ was also observed, which was mainly due to the impact of the change of magnetic anisotropy from the 8:14-epoxy linkage (oxirane) in stauntoside V_3_ to the Δ^8(14)^ structure in **1**. For the rest of carbon atoms, except for C-12 and C-18 which, because of the same causes as C-10, showed up lowfield shifts of Δ_δ_ +1.4 and +1.82, respectively, the numerical range of the absolute values of Δ_δ_ compared with the corresponding carbons in stauntoside V_3_ were less than 1.0, including those of the sugar moieties. These consistencies and differences of functional groups and chemical shifts between **1** and stauntoside V_3_, especially the molecular formula with a hydrogen deficiency index of twelve, indicated the presence of the peroxo bridge structure between C-5 and C-9, just as in stauntoside V_3_. Further, this identification was confirmed by the finding that the peroxylated downfield chemical shifts of Δ_δ_ +11.8 and +11.1 were observed for C-5 and C-9, respectively, in the ^13^C-NMR spectrum compared with stauntoside U, another 14,15-secopregnane-type glycoside with the aglycone of stauntogenin E (8α:14α,14:16,15:20,18:20-tetraepoxy-14,15-secopregn-6-ene-3β,5α,9α-triol) previously isolated by our group from *C. stauntonii* [3]. The HMBC spectrum showed the same picture as those in stauntoside V_3_, with the following correlations being well-marked: H-6 to C-4, 5, 8, and 10; H-7 to C-5, 9, and 14; H-15a to C-16, 17, and 20; H-17 to C-12, 13, 14, 18, and 20; H-18a to C-12 and 14; H-18b to C-12, 14, 17, and 20; Me-19 to C-1, 5, 9, and 10; Me-21 to C-17 and 20; H-1′′′ of α-l-cymaropyranose to C-4′′ of β-d-digitoxopyranose; H-1′′ of β-d-digitoxopyranose to C-4′ of β-d-canaropyranose; and H-1′ of β-d-canaropyranose to C-3, among others (Figure 2). The typical relative configurations of 14,15-secopregnane-type steroids of compound **1**, i.e., both CH_2_-18 and Me-19 in β-orientation and H-16, H-17, and Me-21 all in α-orientation, which was also the same as that of stauntoside V_3_, were affirmed in the NOESY spectrum by the same picture as those of stauntoside V_3_, with NOE correlations of H-3/H-2α, H-3/H-4α, H-6/H-4α, H-6/H-4β, H-6/H-7, H-6/Me-19, H-7/Me-19, H-15α/H-17, H-16/H-17, H-17/Me-21, and Me-19/H-11β being evident and NOE correlations between H-3 and H-1α, H-3 and H-1β, H_2_-18 and Me-21, H_2_-18 and H-17, and Me-19 and H-4α not being observed (Figure 3). Especially, the relative configurations of 5α:9α-peroxy linkage and H-3α were elucidated by the same picture of reciprocal NOE correlations of H-3, H-6, H-7, and Me-19 (Figure 3), among others, with relevant protons as in stauntoside V_3_. Thus, the aglycone was elucidated as 3β-hydroxy-14:16,15:20,18:20-triepoxy-5α:9α-peroxy-14,15-secopregnane-6,8(14)-diene according to the number system of pregnanes and was named as stauntogenin G, and the structure of compound **1** was characterized as 14:16,15:20,18:20-triepoxy-5α:9α-peroxy-14,15-secopregn-6,8(14)-dien-3β-yl-4-*o*-(4-*o*-α-l-cymaropyranosyl-β-d-digitoxopyranosyl)-β-d-canaropyranoside and named stauntoside UA.

Compound **2** possessed a molecular formula of C_49_H_74_O_19_ according to its ^13^C-NMR data and the HRESIMS (positive ion mode) of the sodium adduct molecular ion at *m*/*z* 989.4729, indicating a hydrogen deficiency index of thirteen. Its IR spectrum displayed absorption bands for hydroxy (3393 cm^−1^) and olefinic (1646 cm^−1^) functionalities. Acid hydrolysis of **2**, along with derivatization and GC analysis, indicated the presence of d-thevetopyranose, d-cymaropyranose, and l-diginopyranose in a 1:2:1 ratio. A detailed comparison of the ^1^H- and ^13^C-NMR data between **1** and **2** showed that **2** was nearly entirely identical to **1**, with respect to their aglycone moieties (Table 1 and Table 2), the numerical range of Δ_δ_ for all the ^13^C-NMR signals of the aglycone of **2** compared with the corresponding carbons in **1** was between +1.1 and +1.6, which were obviously systematic errors, suggesting the same aglycone for **2** and **1**. This determination was confirmed with a combined interpretation of the 2D NMR spectra of **2**, including the ^1^H,^1^H-COSY, HSQC, and HMBC correlations (data not shown). In addition to the resonances of the aglycone moiety, the ^1^H and ^13^C-NMR data (Table 1 and Table 2) and the 2D NMR spectroscopic features, including the ^1^H,^1^H-COSY and HMBC correlations, among others, of the sugar moiety of **2** were consistent with stauntoside V_1_, a 14,15-secopregnane-type glycoside previously isolated by our group from *C. stauntonii* [3]. Thus, the structure of **2** is 14:16,15:20,18:20-triepoxy-5α:9α-peroxy-14,15-secopregn-6,8(14)-dien-3β-yl-4-*O*-[4-*O*-(4-*O*-α-l-diginopyranosyl-β-d-cymaropyranosyl)-β-d-cymaropyranosyl]-β-d-thevetopyranoside and named stauntoside UA_1_.

Compound **3** possessed a molecular formula of C_48_H_72_O_19_ according to its ^13^C-NMR data and the HRESIMS (positive ion mode) of the sodium adduct molecular ion at *m*/*z* 975.4567, indicating a hydrogen deficiency index of thirteen. Its IR spectrum displayed absorption bands for hydroxy (3369 cm^−1^) and olefinic (1662 cm^−1^) functionalities. Acid hydrolysis of **3**, along with derivatization and GC analysis, indicated the presence of d-thevetopyranose, d-digitoxopyranose, d-cymaropyranose, and l-diginopyranose in a 1:1:1:1 ratio. A detailed comparison of the ^1^H- and ^13^C-NMR data of **1**–**3** indicated that they shared the same aglycone, compound **3** being nearly entirely identical to **1** and **2**, with respect to their aglycone moieties and the numerical range of Δ_δ_ for all the ^13^C-NMR signals of the aglycone of **3** compared with the corresponding carbons in **2** being between −1.4 and −1.6 (Table 1 and Table 2). This determination was confirmed with a combined interpretation of the 2D NMR spectra of **3**, including the ^1^H,^1^H-COSY, HSQC, and HMBC correlations (data not shown). In addition to the resonances of the aglycone moiety, the ^1^H- and ^13^C-NMR data (Table 1 and Table 2) and the 2D NMR spectroscopic features, including the ^1^H,^1^H-COSY and HMBC correlations, among others, of the sugar moiety of **3** were consistent with stauntoside W, a 14,15-secopregnane-type glycoside previously isolated by our group from *C. stauntonii* [3]. Thus, the structure of **3** is 14:16,15:20,18:20-triepoxy-5α:9α-peroxy-14,15-secopregn-6,8(14)-dien-3β-yl-4-*O*-[4-*O*-(4-*O*-α-l-diginopyranosoyl-β-d-cymaropyranosyl)-β-d-digitoxopyranosyl]-β-d-thevetopyranoside and named stauntoside UA_2_.

## 3. Experimental Section

### 3.1. General Experimental Procedures

All the instruments, solvents, reagents, and experimental conditions for the measurements of IR spectra, 1D and 2D NMR spectra, and both ESIMS and HRESIMS data and for the performing of column chromatography (CC), preparative HPLC procedure, and TLC analysis were previously described [2,3].

### 3.2. Plant Material

The collecting, species identifying, and depositing of the roots of *C. stauntonii* were previously described [2,3].

### 3.3. Extraction and Isolation

The extraction of the dried and pulverized roots (30 kg) of *C. stauntonii* and the preliminary isolation of the 95% EtOH extract and the consequent EtOAc-soluble portion to afford thirteen subfractions (Fr. 1 to Fr. 13) of silica gel CC fractionation were previously described [2,3]. Fr. 3 (68.0 g; eluted with CHCl_3_:MeOH, 100:1, *v*/*v*) was separated with silica gel CC using a gradient elution of petroleum ether:EtOA (25:1→1:1, *v*/*v*) to yield seven subfractions, Fr. 3-1 to Fr. 3-7. Fr. 3-5 (5.0 g; eluted with petroleum ether:EtOAc, 10:1, *v*/*v*) was subjected to a flash C_18_ column eluted with a gradient of MeOH:H_2_O (40:60→100:0, *v*/*v*) to yield six subfractions, Fr. 3-5-1 to Fr. 3-5-6. Fr. 3-5-4 (sampled 150 mg; eluted with MeOH:H_2_O, 60:40, *v*/*v*) was subjected to preparative RP-HPLC (mobile phase of MeCN:H_2_O (38:62, *v*/*v*) at a flow rate of 5 mL min^−1^ with UV detection at 280 nm) to yield, in addition to the reported known compound [3], compound **1** (15 mg). Fr. 5 (12.0 g; eluted with CHCl_3_:MeOH, 50:1, *v*/*v*) was subjected to a flash C_18_ column eluted with a gradient of MeOH:H_2_O (40:60→100:0, *v*/*v*) to yield five subfractions, Fr. 5-1 to Fr. 5-5. Fr. 5-4 (6.0 g; eluted with MeOH:H_2_O, 70:30, *v*/*v*) was subjected to a flash C_18_ column eluted with a gradient of MeOH:H_2_O (40:60→100:0, *v*/*v*) to yield five subfractions, Fr. 5-4-1 to Fr. 5-4-5. Fr. 5-4-4 (sampled 800 mg, eluted with MeOH:H_2_O, 70:30, *v*/*v*) was subjected to preparative RP-HPLC (mobile phase of CH_3_CN:H_2_O (38:62, *v*/*v*) at a flow rate of 5 mL min^-1^ with UV detection at 280 nm) to yield compound **2** (17 mg). Fr. 8 (6.0 g, eluted with CHCl_3_:MeOH, 25:1, *v*/*v*) was subjected to a flash C_18_ column eluted with a gradient of MeOH:H_2_O (40:60→100:0, *v*/*v*) to yield five subfractions, Fr. 8-1 to Fr. 8-5. Fr. 8-2 (sampled 300 mg, eluted with MeOH:H_2_O, 50:50, *v*/*v*) was subjected to preparative RP-HPLC (mobile phase of CH_3_CN:H_2_O (35:65, *v*/*v*) at a flow rate of 5 mL min^−1^ with UV detection at 280 nm) to yield compound **3** (10 mg).

Stauntoside UA (**1**). White amorphous powder; IR (KBr) ν_max_ 3440, 2933, 1681, 1450, 1379, 1163, 1095, 1060, 942, and 835 cm^−1^; for ^1^H-NMR (500 MHz), see Table 1 and Table 2; for ^13^C-NMR (125 MHz), see Table 1 and Table 2; positive-ion mode ESIMS *m*/*z* 801.5 [M + Na]^+^; positive-ion mode HRESIMS *m*/*z* 779.3861 [M + H]^+^ (calculated for C_40_H_59_O_15_, 779.3848), *m*/*z* 801.3694 [M + Na]^+^ (calculated for C_40_H_58_O_15_Na, 801.3668).

Stauntoside UA_1_ (**2**). White amorphous powder; IR (KBr) ν_max_: 3393, 2921, 1646, 1468, 1380, 1160, 1104, 1063, 1007, and 721 cm^−1^; for ^1^H-NMR (500 MHz), see Table 1 and Table 2; for ^13^C-NMR (125 MHz), see Table 1 and Table 2; positive-ion mode ESIMS *m*/*z* 989.5 [M + Na]^+^; positive-ion mode HRESIMS *m*/*z* 989.4729 [M + Na]^+^ (calculated for C_49_H_74_O_19_Na, 989.4717).

Stauntoside UA_2_ (**3**). White amorphous powder; IR (KBr) *ν*_max_: 3369, 2924, 1662, 1465, 1380, 1165, 1065, 1023, and 879 cm^−1^; for ^1^H-NMR (500 MHz), see Table 1 and Table 2; for ^13^C-NMR (125 MHz), see Table 1 and Table 2; positive-ion mode ESIMS *m*/*z* 975.5 [M + Na]^+^; positive-ion mode HRESIMS *m*/*z* 975.4567 [M + Na]^+^ (calculated for C_48_H_72_O_19_Na, 975.4560).

### 3.4. Determination of Steroidal Category and 2-Deoxysugars

#### 3.4.1. Libermann-Burchard Reaction

To a solution of each compound (1 mg) in acetic anhydride (5 mL) was added a little of a mixture of 98% sulfuric acid and acetic anhydride (1:20, *v*/*v*) dropwise. An obvious color change from somewhat yellowish red to purple to blue to dark reddish brown was observed.

#### 3.4.2. Keller-Kiliani Reaction

To a solution of each compound (1 mg) in acetic acid (5 mL) was added one drop of aqueous 20% FeCl_3_ solution. The solution was fully mixed and then a spot of 98% sulfuric acid was added along the test tube wall, with a light green color being observed in the acetic acid solution.

### 3.5. Acid Hydrolysis of New Compounds and Determination of Absolute Configurations of Monosaccharides

The acid hydrolysis of new compounds and determination of absolute configurations of monosaccharides were conducted using the method described in a previous paper from our laboratory [2,16]. In this experiment, the known compounds stauntoside B, glaucogenin C mono-d-thevetoside, stauntoside G, and amplexicoside D were used to determine the retention times of the acetylated thiazolidine derivatives of relevant monosaccharides, with *t*_R_ d-digitoxose 13.09 min, *t*_R_ l-cymarose 13.46 min, *t*_R_ d-cymarose 18.46 min, *t*_R_ l-diginose 14.31 min, *t*_R_ d-thevetose 16.07 min, and *t*_R_ d-canarose 16.51 min being determined. Retention times of the monosaccharides released from the new compounds after derivatization were as follows: *t*_R_ d-digitoxose 13.05 min, *t*_R_ l-cymarose 13.41 min, and *t*_R_ d-canarose 16.45 min for compound **1**; *t*_R_ d-cymarose 18.49 min, *t*_R_ l-diginose 14.38 min, and *t*_R_ d-thevetose 16.09 min for compound **2**; and *t*_R_ d-digitoxose 13.06 min, *t*_R_ d-cymarose 18.42 min, *t*_R_ l-diginose 14.38 min, and *t*_R_ d-thevetose 16.03 min for compound **3**.

## Figures and Tables

**Table 1:** ^1^H- and ^13^C-NMR spectroscopic data for the aglycone moieties of **1**–**3** (in pyridine-*d*_5_, TMS).

Position	1		2		3	
	δ_H_ (*J* in Hz)	δ_C_	δ_H_ (*J* in Hz)	δ_C_	δ_H_ (*J* in Hz)	δ_C_
1α	1.34, ov	27.6	1.32, ov	28.9	1.32, ov	27.4
1β	2.10, ov		2.10, ov		1.91, ov	
2α	2.23, br dd (14.0, 2.5)	30.2	2.39, ov	31.4	2.23, ov	29.9
2β	2.08, ov		2.10, ov		2.07, ov	
3	4.31, m	73.3	4.23, m	74.9	4.31, m	73.4
4α	2.51, ov	33.6	2.52, dd (14.0, 4.5)	34.7	2.52, dd (14.0, 5.0)	33.2
4β	1.96, ov		1.75, ov		1.76, ov	
5		85.6		86.8		85.3
6	5.59, d (9.5)	129.1	5.48, d (9.5)	130.3	5.48, d (9.5)	128.8
7	6.77, d (9.5)	125.1	6.72, d (9.5)	126.4	6.72, d (9.5)	124.8
8		110.4		111.7		110.1
9		87.4		88.6		87.1
10		50.6		51.9		50.4
11α	2.01, ov	24.7	2.00, ov	25.9	2.00, ov	24.4
11β	1.77, ov		1.72, ov		1.75, ov	
12a	2.10, ov	28.4	2.01, ov	29.7	2.08, ov	28.2
12b	1.92, ov		1.92, ov		1.91, ov	
13		55.0		56.2		54.7
14		156.8		158.1		156.6
15α	3.79, dd (11.0, 4.5)	72.0	3.79, dd (11.0, 4.5)	73.2	3.79, dd (11.0, 4.5)	71.7
15β	4.24, ov		4.24, br d (11.0)		4.09, br d (11.0)	
16	4.81, ov	86.9	4.81, ov	88.2	4.81, ov	86.7
17	2.81, d (7.5)	61.9	2.80, d (8.0)	63.1	2.80, d (8.0)	61.6
18a	3.99, d (9.0)	75.3	3.98, d (10.0)	76.5	3.98, d (8.5)	75.0
18b	4.11, d (9.0)		4.10, d (10.0)		4.09, d (8.5)	
19	0.95, s	15.8	0.89, s	17.0	0.89, s	15.5
20		118.4		119.6		118.2
21	1.54, s	22.4	1.54, s	23.7	1.54, s	22.2

ov: overlapped signals.

**Table 2:** ^1^H- and ^13^C-NMR spectroscopic data for the sugar moieties of **1**–**3** (pyridine-*d*_5_).

Position	1		2		3	
	δ_H_ (*J* in Hz)	δ_C_	δ_H_ (*J* in Hz)	δ_C_	δ_H_ (*J* in Hz)	δ_C_
	β-d-can		β-d-the		β-d-the	
1′	4.76, dd (9.5, 2.0)	98.8	4.74, d (8.0)	103.9	4.73, d (8.0)	102.4
2′a	2.51, ov	40.1	3.91, ov	75.9	3.91, ov	74.4
2′b	1.96, ov					
3′	3.95, ov	70.1	3.66, ov	86.1	3.67, ov	85.6
4′	3.28, ov	88.5	3.67, ov	83.9	3.69, ov	82.6
5'	3.47, ov	70.9	3.61, ov	72.9	3.69, ov	71.4
6′	1.31, d (6.5)	18.1	1.42, d (5.5)	19.9	1.42, d (6.0)	18.4
3′-OCH_3_			3.92, s	61.8	3.93, s	60.3
	β-d-digt		β-d-cym		β-d-digt	
1′′	5.24, dd (9.5, 2.0)	99.9	5.30, dd (10.0, 2.0)	100.1	5.55, dd (10.0, 2.0)	98.7
2′′a	2.41, ov	38.2	1.85, ov	38.4	1.99, ov	38.9
2′′b	1.96, ov		2.35, ov		2.42, ov	
3′′	4.48, ov	67.4	4.07, ov	79.4	4.63, ov	67.5
4′′	3.43, ov	80.7	3.48, dd (10.0, 3.0)	84.5	3.47, ov	83.0
5′′	4.19, ov	69.4	4.21, ov	70.6	4.29, ov	68.6
6′′	1.31, d (6.5)	18.1	1.37, d (6.0)	19.9	1.41, d (6.0)	18.3
3′′-OCH_3_			3.61, s	60.1		
	α-l-cym		β-d-cym		β-d-cym	
1′′′	5.04, dd (4.0, 3.0)	98.6	5.08, dd (9.5, 1.5)	101.5	5.13, br d (9.5)	99.4
2′′′a	2.34, ov	32.2	1.74, ov (2.42)	36.4	1.68, ov	34.7_2_
2′′′b	1.82, ov		2.42, ov		2.32, ov	
3′′′	3.79, ov	76.5	3.96, ov	78.8	3.92, ov	77.2
4′′′	3.61, ov	72.6	3.45, dd (10.0, 1.0)	83.5	3.39, ov	82.0
5′′′	4.48, ov	67.6	4.21, ov	70.6	4.21, ov	69.1
6′′′	1.41, d (6.5)	18. 4	1.37, d (6.0)	19.7	1.30, d (6.0)	18.4
3′′′-OCH_3_	3.37, s	56.8	3.52, s	58.6	3.52, s	57.1
			α-l-dign		α-l-dign	
1′′′′			5.22, br d (3.0)	102.4	5.19, br d (3.5)	101.0
2′′′′a			2.09, ov	32.2	2.07, ov	30.7
2′′′′b			2.39, ov		2.37, ov	
3′′′′			3.85, ov	77.1	3.84, ov	75.6
4′′′′			4.07, ov	68.9	4.07, ov	67.4
5′′′′			4.32, ov	68.8	4.30, ov	67.5
6′′′′			1.57, d (6.5)	19.0	1.56, d (7.0)	17.5
3′′′′-OCH_3_			3.31, s	56.3	3.31, s	54.8

ov: overlapped signals.
